# A Compact Two-Loudspeaker Virtual Sound Reproduction System for Clinical Testing of Spatial Hearing With Hearing-Assistive Devices

**DOI:** 10.3389/fnins.2021.725127

**Published:** 2022-01-28

**Authors:** Eric C. Hamdan, Mark D. Fletcher

**Affiliations:** ^1^University of Southampton Auditory Implant Service, University of Southampton, Southampton, United Kingdom; ^2^Institute of Sound and Vibration Research, University of Southampton, Southampton, United Kingdom

**Keywords:** hearing impairment, speech in noise (SIN), sound localization, binaural, clinical audiology, transaural, bilateral, sound field control

## Abstract

Exciting developments in hearing aid and cochlear implant technology for linking signal processing across the ears have improved spatial hearing outcomes. This has resulted in an increased emphasis on clinical assessment of the spatial hearing abilities of hearing-assistive device users. Effective assessment of spatial hearing currently requires a large and costly loudspeaker array system, housed in a heavily acoustically treated testing room. This imposes economic and logistical constraints that limit proliferation of array systems, particularly in developing nations. Despite their size and cost, the ability of current clinical array systems to reproduce realistic spatial sound fields is limited, which substantially reduces the range of realistic acoustic scenes that can be used for diagnostic testing. We propose an alternative low-cost, compact virtual acoustics system with just two loudspeakers. This system uses crosstalk cancelation to reproduce pressure signals at the device microphones that match those for real-world sound sources. Furthermore, in contrast to clinical array systems, the system can adapt to different room acoustics, removing the requirement for a heavily acoustically treated testing environment. We conducted a proof-of-concept study in two stages: in the first, we evaluated the physical performance of the system for a stationary listener in anechoic conditions and in a small audiological testing booth with moderate acoustic treatment. To do this, a head and torso simulator was fitted with specially adapted hearing-assistive devices that allowed direct access to the microphone signals. These microphone signals were compared for real and virtual sound sources at numerous source locations. In the second stage, we quantified the system’s robustness to head rotations with and without the system adapting for head position. In the stationary case, the system was found to be highly effective at reproducing signals, such as speech, at all tested source locations. When head rotation was added, it performed well for rotations of up to 2°, even without adapting. However, performance improved markedly for larger rotations when the system adapted. These findings suggest that a compact, low-cost virtual acoustics system can give wider access to advanced and ecologically valid audiological testing, which could substantially improve clinical assessment of hearing-assistive device users.

## Introduction

Bilateral cochlear implant and hearing aid technology has the potential to restore binaural hearing to hearing-impaired listeners. Binaural hearing is critical for locating and separating sounds, such as speech in noisy environments (Litovsky et al., 2004; Brown and Balkany, 2007; Lovett et al., 2010). However, the signal processing used in hearing-assistive devices (HADs) often distorts interaural level and time differences between the ears (Pastore et al., 2021), which are the primary spatial hearing cues. As a result, many HAD users have limited spatial hearing capabilities (Dorman et al., 2016; Fletcher et al., 2020a; Pastore et al., 2021). While there is a growing interest in approaches for improving spatial hearing in hearing-impaired listeners (e.g., Moore et al., 2016; Williges et al., 2018; Fletcher and Zgheib, 2020; Fletcher et al., 2020a,b; Gajecki and Nogueira, 2021), clinical testing of spatial hearing ability remains limited.

There are currently several sound field reproduction methods for assessing spatial hearing abilities of HAD users and the directional processing capabilities of HADs. The most common method is to play back sounds using a spatially distributed array of loudspeakers (Seeber et al., 2004, 2010; Lovett et al., 2010; Kitterick et al., 2011). The loudspeakers are typically arranged in a circle or semicircle around the listener, as in the Crescent of Sound system that is used clinically across the United Kingdom (Kitterick et al., 2011). Because these systems use simple direct-speaker playback or amplitude panning, the sound that reaches the ears can be colored by the acoustics of the room in which the system is housed. The room should therefore be heavily acoustically treated to ensure that system performance is equivalent across clinics. However, this is rarely achieved and systems such as the Crescent of Sound do not have an operating standard for the acoustic treatment of the room they are used in. Furthermore, because the method only allows reproduction of sound sources from a limited set of locations, these systems are unable to accurately reproduce complex auditory scenes that are typically encountered in the real world. This limits the ecological validity of the tests that can be performed. In addition, systems with many loudspeakers, such as the Crescent of Sound, are expensive and need to be housed in a large room. This severely limits proliferation, particularly in low- and middle- income countries.

An alternative to current clinical array systems are virtual acoustics (VA) systems. These seek to simulate the perception of real-world spatial sounds and include a variety of approaches (Lokki and Savioja, 2008). Previously proposed VA systems have used techniques such as higher-order ambisonics and vector base amplitude panning (VBAP) in combination with large loudspeaker arrays, e.g., more than 20 loudspeakers (Minnaar et al., 2013; Grimm et al., 2015, 2016; Cubick and Dau, 2016; Oreinos and Buchholz, 2016). Loudspeaker arrays of this size are impractically large and expensive, and seen as not suitable for clinical use. However, higher-order ambisonics constrained to the horizontal plane would only require (2*N* + 1) loudspeakers, where *N* is the order (Zotter and Frank, 2019). Still, the practicality of ambisonics systems could be limited as they require more loudspeakers in exchange for higher accuracy offered by higher orders of reproduction. More recently, Meng et al. (2021) investigated a smaller two-loudspeaker VBAP system for facilitating a minimum audible angle test. However, the virtual source positioning and reproduction accuracy are limited, as VBAP restricts the position of the virtual source to within the span of the loudspeakers. Furthermore, horizontal plane VBAP is not designed to faithfully reproduce sources above and below the listener. This means that the system is substantially limited in its ability to produce realistic acoustic scenes.

Aside from these VA methods, there are binaural methods for evaluating bilateral HADs. Headphones placed over the devices are a common approach to delivering binaural audio. However, headphones can be obtrusive and binaural signals delivered through headphones are most often derived from binaural recordings (i.e., microphones placed at the opening of the ear canals) or binaural synthesis [i.e., simulated using measured head-related transfer functions (HRTFs)]. Neither of these approaches match the pressure signals that would arrive at the HAD microphones in the real world because they are not derived from HAD-related transfer functions. Another possible issue is inconsistent coupling of the headphone loudspeaker with the HAD microphones across headphone fittings, which could further compromise the integrity of the reproduction.

Pausch et al. (2018) used custom-made research hearing aids that allow the microphones to be bypassed and hearing-aid-related binaural signals to be delivered directly to the devices. The research hearing aids were used in tandem with a crosstalk cancelation (CTC) system for reproducing HRTF-based binaural signals at the ear drums. This meant that stimulation was provided for residual hearing as well as through the HAD. However, they did not demonstrate that their CTC system could reproduce accurate target physical pressure signals, making it difficult to evaluate the success of their system. While they reported the channel separation (see section “Metrics”) achieved by their system, this metric alone is insufficient for determining the physical accuracy of the reproduction and ruling out audible artifacts that could diminish perceptual outcomes.

Another approach that used direct input to the HADs was proposed by Chan et al. (2008). In this approach, the binaural signals were calibrated and synthesized using transfer functions measured between a loudspeaker and the HADs when mounted on a dummy head. Measurements were made using either the onboard HAD microphones or separate microphones placed near to the HAD microphones. It is possible that, in the future, device manufacturers or a dedicated service could provide clinicians with transfer functions for each of their devices. However, because microphones are bypassed with the direct input approach, this would mean that defective microphones or changes in microphone response over its lifetime would not be accounted for. Alternatively, sound field measurements could be repeated for each device in clinic. However, the measurements would then be susceptible to local room acoustics, meaning they could act as a significant source of variance between clinical measurements. The protracted calibration process would require additional clinician training and may be unsuitable for clinical appointments, where time is typically limited. Furthermore, this would require the use of potentially expensive additional equipment (e.g., a head and torso simulator).

In the current study, we investigated a VA system that uses two loudspeakers. The system utilizes a type of sound field control based on inverse filters, more commonly known as CTC (e.g., Xie, 2013), designed using HAD transfer functions measured *in situ*. We propose that, when given access to the HAD microphone signals in the clinic environment, signal processing steps can be taken that allow rapid transfer function measurement and inverse filter design. These inverse filters enable pressure signal reproduction at the device microphones. Such a system could precisely control the sound field at the device and allow the reproduction of complex real-world auditory scenes, while remaining unobtrusive. Inverse filters could also allow a room agnostic approach, where a measurement standard can be retained across clinical settings. Furthermore, because the system only requires two loudspeakers, it would be inexpensive and have a small physical footprint. The compactness, low cost, room adaptability, and capacity to accommodate tests with high ecological validity would give this system major advantages over current clinical systems.

The first aim of the study was to assess the ability of the system to reproduce physically accurate sound fields at the HAD microphones for a stationary listener. We evaluated the sound fields by measuring and analyzing the sound pressure at the HAD microphones. These measurements were taken in both an ideal (anechoic) and a representative clinical environment. The reproduced sound pressure was analyzed using several metrics that directly measure the system’s performance, both in the frequency and time domains. From these metrics, we establish a baseline standard (that does not currently exist) for what sound field control can be physically achieved at the HAD microphones. We set a target channel separation of at least 20 dB between the reproduced left and right binaural signals measured at the HAD microphones. This amount of channel separation has been shown to be the minimum amount needed to give equivalent perception of the original and reproduced binaural signal in normal-hearing listeners (e.g., Parodi and Rubak, 2011). We demonstrated that our system is capable of achieving this target channel separation in both listening environments and that the time domain error was low. In this first part of the experiment, we demonstrated that, for a stationary listener, the system can reproduce target HAD microphone signals to a high degree of accuracy.

The second aim of the study was to assess the impact of minor involuntary head movements (“postural sway”), that are likely to occur in clinic even if participants are instructed to sit still (e.g., Hirahara et al., 2010; Denk et al., 2017). Using the aforementioned metrics, we compared the system performance with and without compensating for head movements to establish whether head movement compensation is required. We show that the system is robust to the small head rotations (of 2° or less) that would be expected in clinical assessments, and that good performance can be achieved for head rotations up to 10° if the system adapts for changes in head position.

## Materials and Methods

### Test Apparatus

**FIGURE 1:**
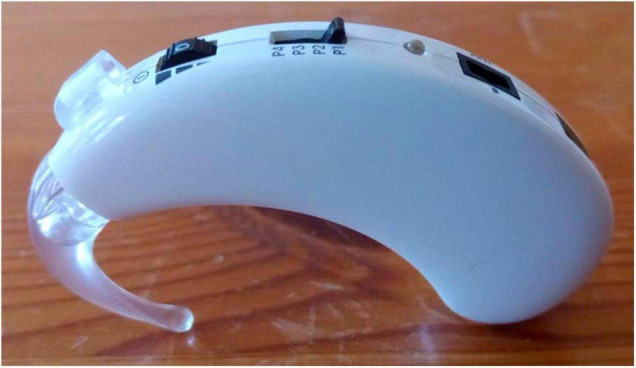
Modified behind-the-ear HADs.

The loudspeakers used were Genelec 8020Cs (Genelec Oy, Iisalmi, Finland). A KEMAR head and torso simulator (G.R.A.S. Sound and Vibration, Holte, Denmark) was fitted with custom HADs behind each ear to simulate a stationary seated listener. The KEMAR was placed on a stand to allow easy placement and rotation. The HADs we used were modified Oticon Medical (Smørum, Denmark) Saphyr CI processors (TX9 model; shown in Figure 1), with an onboard sampling rate of 16 kHz. The modification allowed direct access of the microphone signals via analog cable outputs so that the sound field could be controlled at the microphones directly. All onboard signal processing was bypassed. For recording the HAD microphone signals, the analog outputs of the microphones were sent to an RME UC (RME Audio, Haimhausen, Germany) for digital conversion. The digital signals were sent from the RME UC via USB to a computer running the measurement and reproduction software. A custom Matlab (version R2020b, The MathWorks, Inc., Natick, MA, United States) script was written to record the microphone signals to the computer during measurements. For loudspeaker reproduction, the same RME UC was used to output audio signals to the loudspeaker array. All audio playback was done with either Matlab or Max/MSP (Version 8.1.2, Cycling ’74, Walnut, CA, United States). All signals were recorded and reproduced at a sampling rate of 48 kHz with a bit depth of 24 bits.

In order to measure head rotation, a HTC VIVE tracker (Version 1, HTC Corporation, Xindian, New Taipei, Taiwan) was fitted to the top of the KEMAR head with a plastic cap in between measurements. The head tracker was removed before taking a new measurement.

### Testing Environments

**FIGURE 2:**
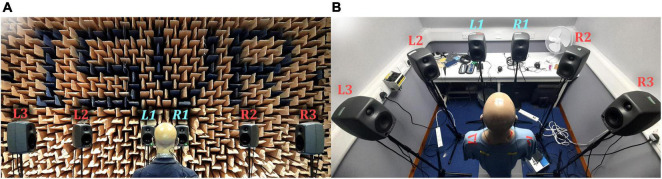
Photographs of the reproduction setups: **(A)** Anechoic chamber; **(B)** Clinical booth. L1 and R1 (labeled in light blue) were used for the VA system.

Two testing environments were used: the Institute of Sound and Vibration Research (University of Southampton, United Kingdom) anechoic chamber (shown in Figure 2A) and a clinical audiological testing booth (shown in Figure 2B) located in the Hearing and Balance Centre (University of Southampton, United Kingdom). The anechoic chamber was chosen to represent an ideal testing environment with heavy acoustic treatment. The clinical booth was chosen to represent a typical clinical testing environment. The test booth was 2.5 m by 2.1 m, with a ceiling height of 2.05 m and had a background noise level conforming to the recommendation of British Society of Audiology (2017).

### Sound Field Reproduction

The reproduction system consisted of a six-channel loudspeaker array arranged in a semicircle, with the KEMAR positioned at the center of the semicircle facing the center of the array (see Figure 2). Each loudspeaker was placed 1.5 m in the anechoic chamber and 1 m in the booth (due to space constraints) from the center of the KEMAR head and set at ear height. All loudspeakers were used to produce reference signals, to compare against virtual sources. Loudspeakers L1 and R1 (labeled in light blue in Figure 2) were chosen for the VA system to retain a compact array.

### Pressure Matching Method

The method of sound field control underlying the VA system was the Tikhonov regularized pressure matching method (e.g., Kirkeby et al., 1998; Olivieri et al., 2015). When applied to just two loudspeakers, the pressure matching method has been more commonly known as CTC (see Xie, 2013 for an overview of CTC technology). The overall sound field control problem is described in the frequency domain as:


$$\text{G}\,\left(\omega \right)\,\text{q}\,\left(\omega \right)\,\overset{!}{=}d\,\left(\omega \right),$$

where $\overset{!}{=}$ means ‘ideally equal to’; ***d***(ω) = [*d*_1_(ω) *d*_2_(ω)]^*T*^ ∈ ℂ^2^ is the vector of target pressure signals that we wish to reproduce at two HAD microphones; the so-called “plant matrix”:


$$\text{G}\,\left(\omega \right)=\left[\begin{array}{c} {\text{g}}_{1}^{T}\,\left(\omega \right)\\ {\text{g}}_{2}^{T}\,\left(\omega \right) \end{array}\right]\in {\mathbb{C}}^{2\times 2},$$

is composed of electroacoustic transfer functions between the two microphones and two loudspeakers; ***g***_*m*_(ω) = [*g*_*m*1_(ω) *gm*_2_(ω)]^*T*^ ∈ ℂ^2^ is the *m*th vector of electroacoustic transfer functions between the *m*th microphone and each loudspeaker, where *m* = 1, 2; and ***q***(ω) = [*q*_1_(ω) *q*_2_(ω)]^*T*^ ∈ ℂ^2^ is the vector of *unknown* loudspeaker signals that we wish to calculate. Note that ω is radian frequency. When we apply a calculated set of loudspeaker signals, the physical result is:


$$\text{G}\,\left(\omega \right)\,{\text{q}}_{0}\,\left(\omega \right)=\text{p}\,\left(\omega \right),$$

where ***p***(ω) = [*p*_1_(ω) *p*_2_(ω)]^*T*^ ∈ ℂ^2^ is the vector of reproduced pressure signals at the microphones, having applied a specific set of loudspeaker signals ***q***_0_(ω). The desired result of the system, can be expressed as:


$$\text{p}\,\left(\omega \right)=\text{d}\,\left(\omega \right)\,e^{-j\,\omega \,\tau },$$

where τ is a delay in seconds. This means that the reproduced pressure signals are an exact, delayed copy of the target pressure signals. To obtain an inverse filter solution, the Tikhonov regularized inverse solution was calculated using the well-known equation:


$${\text{q}}_{0}\,\left(\omega \right)={\text{G}}^{H}\,\left(\omega \right)\,{\left(\text{G}\,\left(\omega \right)\,{\text{G}}^{H}\,\left(\omega \right)+\beta \,\text{I}\right)}^{-1}\,\text{d}\,\left(\omega \right),$$

where β is the real-valued regularization parameter (here frequency-independent); ***I*** is a 2×2 identity matrix; and (⋅)^−1^ denotes the matrix inverse. Note that in our experiments, the number of microphones was always equal to or less than the number of loudspeakers. In practice, due to the need to regularize (non-zero β) and truncate the inverse filters, an exact solution is generally impossible. However, using this approach allowed filter stability to be obtained.

### Inverse Filter Design

The inverse filter design was divided into two primary stages: first, the *in situ* hearing-assistive device transfer function (HADRTF) measurements and, second, the inverse filter calculation. For the HADRTF measurements, we used an exponential sine sweep as proposed by Farina (2000). An exponential sine sweep from 1 Hz to 24 kHz was played from each loudspeaker and simultaneously recorded at the device microphones. The hearing-assistive device impulse responses (HADIRs) were extracted from each measurement. The frequency domain equivalents of these HADIRs were the HADRTFs used for the inverse filter design.

**FIGURE 3:**
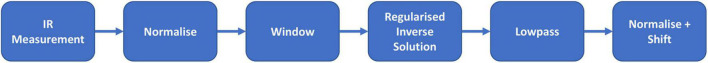
Block diagram showing the inverse filter design process.

After the HADIRs were obtained, the process outlined in Figure 3 was used to generate the inverse filters. The first step was to normalize the HADIRs to take full advantage of the available digital dynamic range. The second step was to temporally window the HADIRs to remove later reflections and noise that can cause instability in the inverse filters. We used a modified Tukey window consisting of concatenated raised cosine sections with a flat rectangular window section. This modification allowed for tuneable fade in and out positions and window length. It was found that a balance of temporal windowing and regularization was needed to produce stable filters and accurate performance, in both spaces. The windowed HADIRs were then converted to the frequency domain to obtain the final HADRTFs. For each frequency bin, the HADRTFs were used to populate the plant matrix. Next, the inverse filters were constructed from the Tikhonov regularized pseudoinverse of the plant matrix using a regularization value of β = 0.0005 for the anechoic chamber and β = 0.001 for the clinical booth. The frequency domain inverse filters were converted to time domain filters. The resulting time domain inverse filters were low-pass filtered with a linear phase FIR filter (99 taps and cutoff frequency at 8000 Hz) to reduce instabilities due to a roll-off in the HADIR magnitude responses around the Nyquist frequency. Following this, the time domain inverse filters were normalized to a peak amplitude of 1 to reduce the need for further amplification at the loudspeaker stage. Lastly, the final time domain inverse filters were shifted to ensure causality and sufficient decay before and after the main peak of each filter. This helped to ensure that time domain artifacts, such as smearing and echoes, were avoided. Full details of the computation applied is provided in the Supplementary Material.

### Sound Field Evaluation

The VA system’s physical performance was evaluated based on its ability to reproduce a target binaural signal in each testing environment. This included assessment of the VA system’s ability to reproduce sources from spatial positions well away from the immediate vicinity of the VA loudspeakers. Here, we highlight the performance when the target signal originated from loudspeaker L3 (see Figure 2), which was positioned 90° to the left of the KEMAR. We assessed the VA system’s room adaptation potential by attempting to reproduce an anechoic signal in the clinical booth. Additionally, we evaluated the system’s robustness to minor head rotations with and without adaptation of the inverse filters for the head movement. We limited the study to head rotations of no more than 10° to the left and right, at single degree increments (positive angles were to the right and negative angles were to the left of center). HADIR measurements were taken for each head rotation angle, in each room, and corresponding inverse filters were calculated from these measurements using the same parameters as the static filters (detailed in section “Inverse Filter Design”). We measured the reproduced performance when the head was rotated using inverse filters designed only for the center position (0°), thus evaluating the system’s robustness without compensation for head rotation. Additionally, for each head rotation position, performance measurements were made with the inverse filters constructed from the HADIRs at that given head rotation angle (i.e., with the head rotation accounted for).

To create target microphone recordings in the anechoic chamber, a recorded female speech sample (Fletcher et al., 2020a; available at DOI: 10.5258/SOTON/D1206) was played from each loudspeaker successively and recorded by the front HAD microphones, so that six stereo HAD recordings of the speech played from the six loudspeaker positions were obtained. These recordings were then filtered with the same low-pass filter applied to the inverse filters to ensure that any energy beyond the HAD Nyquist frequency (8 kHz) was negligible. The target binaural signals were convolved with the inverse filters in real-time and the resulting loudspeaker signals were played back over the VA loudspeakers. The resulting HAD microphone signals were recorded and compared to the target HAD recordings using the time domain error metrics detailed in the following section. Before comparison, the reproduced recordings were time aligned with the target (removal of the constant modeling and lowpass delays) and all recordings were cropped to 1.6 s (76,800 samples at the recording sampling rate of 48 kHz) to remove unnecessary silence.

### Metrics

For the sound field control to work successfully, channel separation must be maintained between the microphones. Additionally, a signal desired at either of the microphones should be as uncolored as possible by the reproduction method itself. Therefore, channel separation alone is insufficient for accurate reproduction. To estimate these qualities, we calculated the left and right microphone signals, *p*_1_(ω), *p*_2_(ω), respectively, after applying the inverse filters. The target signals were unit impulses to the left and right input channels (one at a time) while sending zeros to the other channel, i.e.,


$$d\left(\omega \right)={\left[\begin{array}{cc} 1 & 0 \end{array}\right]}^{T},$$

$$d\left(\omega \right)={\left[\begin{array}{cc} 0 & 1 \end{array}\right]}^{T}.$$

For these quantities, the measured HADRTFs between loudspeakers L1 and R1 and the HAD microphones were convolved with the loudspeaker signals (with inverse filters applied to the input) for the forward calculation. We examined the magnitude and phase responses of *p*_1_(ω), *p*_2_(ω) to give an indication of any unwanted artifacts imposed by the inverse filters. Additionally, the resulting frequency-dependent channel separation in each of these cases was measured by:


$$C\,S_{1}\,\left(\omega \right)=\left|\frac{p_{1}\,\left(\omega \right)}{p_{2}\,\left(\omega \right)}\right|,$$

$$C\,S_{2}\,\left(\omega \right)=\frac{1}{C\,S_{1}},$$

respectively.

The ability to reproduce spatial sounds was assessed by attempting to reproduce a target HADRTF due to each loudspeaker. Additionally, the time domain waveforms of the target microphone signals and reproduced microphone signals were compared using the absolute error:


$$A\,E_{1}\,\left[n\right]=\left|d_{1}\,\left[n\right]-p_{1}\,\left[n\right]\right|,$$

$$A\,E_{2}\,\left[n\right]=\left|d_{2}\,\left[n\right]-p_{2}\,\left[n\right]\right|,$$

where *p*_1_[*n*], *p*_2_[*n*] and *d*_1_[*n*], *d*_2_[*n*] are the discrete time domain reproduced and target signals at microphones 1 and 2 (front left and right), respectively, and *n* is the time sample index. For visual presentation, the absolute errors were presented with smoothing applied from a Savitzky–Golay filter with window length 1001 and polynomial order 1. Additionally, the mean absolute error (MAE) of each channel was also evaluated and calculated as:


$$M\,A\,E_{1}=\frac{\sum_{n=0}^{N-1}A\,E_{1}\,\left[n\right]}{N}$$

$$M\,A\,E_{2}=\frac{\sum_{n=0}^{N-1}A\,E_{2}\,\left[n\right]}{N},$$

where *N* is the total number of samples in each time domain recording (here *N=76800*).

Note that dB quantities in this work were calculated as 20*log*_10_⁡*x*, where *x* is an amplitude quantity in linear scale.

## Results

### Stationary Measurements

For both the anechoic chamber and clinical booth, inverse filters were created for loudspeakers L1 and R1 (see Figure 2) according to the procedure detailed in section “Inverse Filter Design.” The head and torso simulator was kept in a forward-facing position centered between the two loudspeakers for all measurements. The achieved channel separation and ability to reproduce a target HADRTF and time domain waveform was evaluated according to the procedure detailed in section “Metrics.” The VA system’s room adaptation potential was assessed by reproducing anechoic recordings in the booth setting.

#### Anechoic Chamber

**FIGURE 4:**
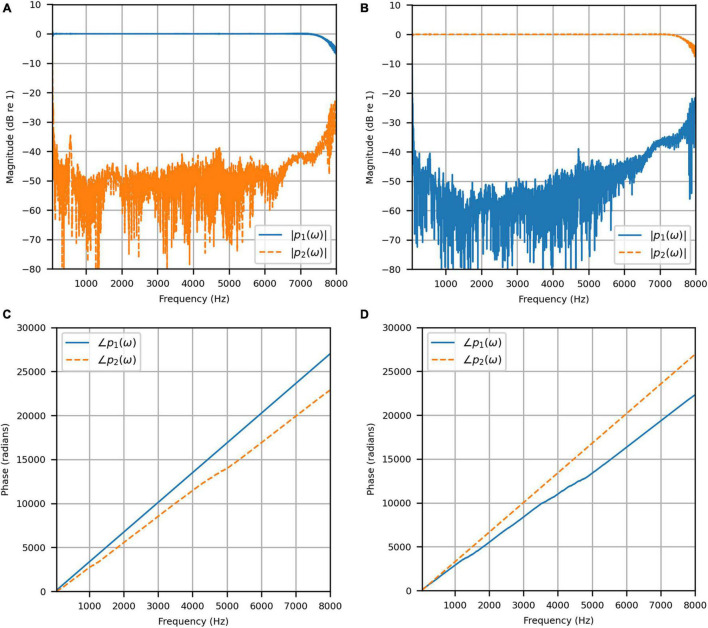
Anechoic chamber: **(A)** Reproduced magnitude responses for a target impulse to the left microphone (blue solid); **(B)** Reproduced magnitude responses for a target impulse to the right microphone (red dashed); **(C)** Reproduced phase responses for a target impulse to the left microphone (blue solid); **(D)** Reproduced phase responses for a target impulse to the right microphone (red dashed).

**FIGURE 5:**
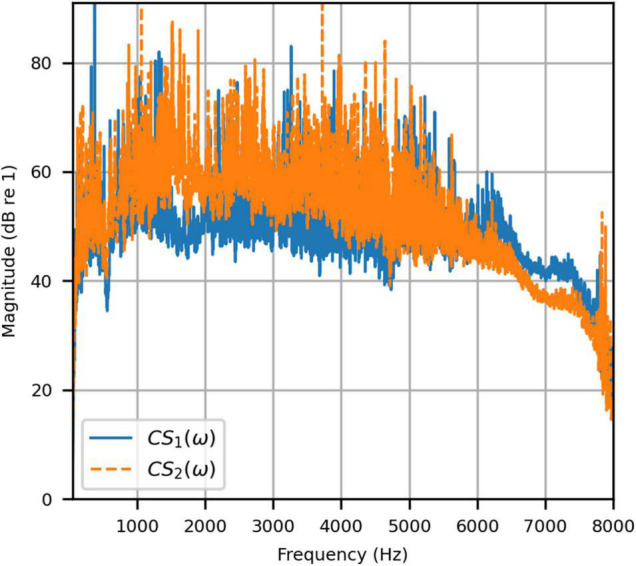
Anechoic chamber: Channel separations for a target impulse to the left (blue solid) and right (red dashed) microphones.

Figure 4 shows the reproduced magnitude responses |*p*_1_(ω)|,|*p*_2_(ω)| and the corresponding unwrapped phase responses ∠*p*_1_(ω),∠*p*_1_(ω), as functions of frequency in Hz for frequencies 50–8000 Hz, as a result of a target impulse to the left and right binaural input channels, respectively. For each channel, the impulse signal was reproduced with an almost flat magnitude response centered around 0 dB, with fluctuations less than 0.5 dB throughout the passband. There was a roll-off around 55 Hz due to regularization, however, these low frequencies are unimportant in most practical use cases. This result confirms that the target signal magnitude responses can be well reproduced. In each case, the opposite channel, i.e., the side where zero pressure was desired, was substantially attenuated. Figure 5 shows that the achieved channel separations *CS*_1_(ω), *CS*_2_(ω) were never less than 40 dB from around 100–6500 Hz (except for the slightly less amount of 39 dB around 4700 Hz), and no less than 20 dB to the limit of the effective passband (7800 Hz) where the applied lowpass filter had already taken substantial effect. The phase responses were essentially linear in the passband.

**FIGURE 6:**
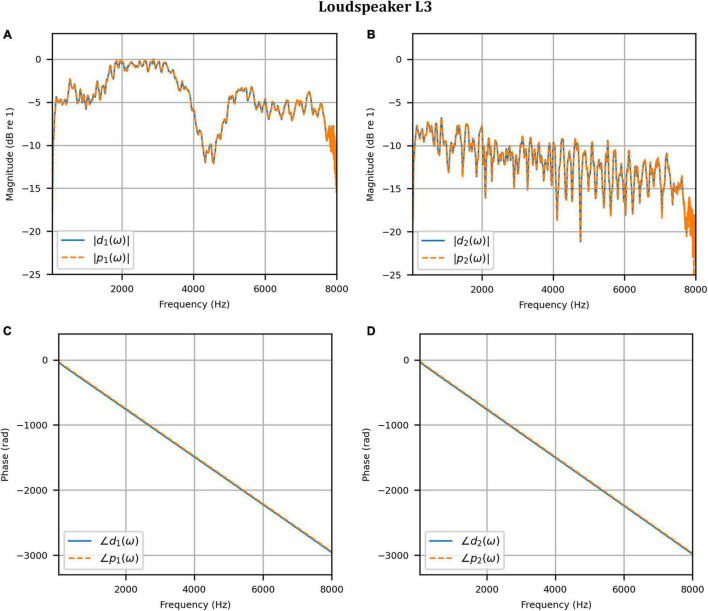
Anechoic chamber: Target (blue solid) versus reproduced (red dashed) frequency responses for the HADRTF due to loudspeaker L3: **(A)** Magnitude responses at the left microphone; **(B)** Magnitude responses at the right microphone; **(C)** Phase responses at the left microphone; **(D)** Phase responses at the right microphone.

**FIGURE 7:**
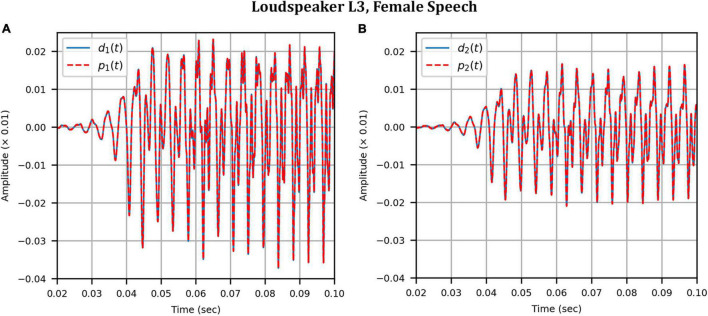
Anechoic chamber: Target (blue solid) versus reproduced (red dashed) time domain waveforms of a female speech sample played from loudspeaker L3: **(A)** Left microphone; **(B)** Right microphone.

**FIGURE 8:**
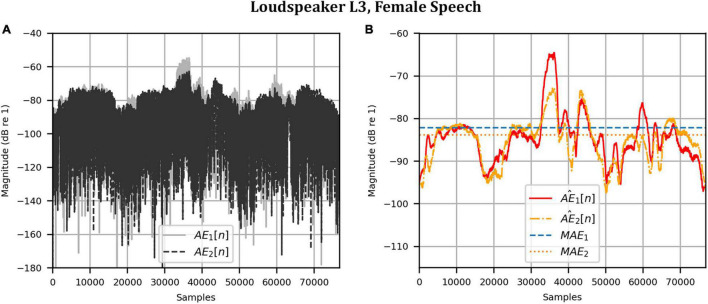
Anechoic chamber: Error metrics quantifying the difference between the target and reproduced time domain waveforms in Figure 7: **(A)** Unsmoothed absolute errors; **(B)** Smoothed absolute errors and mean absolute errors.

Next, the target binaural signal was set to the HADRTFs measured from each loudspeaker. Figure 6 shows the reproduced magnitude and phase response at the left and right HAD microphones when the target was the HADRTF due to loudspeaker L3. There was excellent agreement between the target and reproduced magnitude responses in the passband, with only minor fluctuations at the lowest frequencies. The reproduced phase responses (with the constant modeling delay removed) were also in excellent agreement, although a small constant shift was seen in each channel, thus the original phase relationships between microphones were retained. Excellent agreement between reproduced and target responses was also observed for the remaining loudspeakers (including when the HADRTF was from either of the VA loudspeakers). However, it was important to verify the performance in the time domain and to assess the results in subjective listening tests. The front HAD microphone recordings of the female speech sample played from each loudspeaker were compared to the VA system’s reproduction of that same recording using loudspeakers L1 and R1. Figure 7 compares a portion of the recorded time domain waveforms when speech originated from loudspeaker L3 (the furthest from the VA speakers). With the constant delay removed, excellent alignment of the time domain waveforms is observed. Overall, as expected from the HADRTF reproduction, we found that the agreement in the time domain reproduction was excellent for all six loudspeakers. Figure 8A shows the corresponding AEs (unsmoothed) and Figure 8B shows the smoothed AEs and MAEs, all in dB. Smoothed versions of the AEs are denoted by ${\hat{A\,E}}_{1}\,\left[n\right],{\hat{A\,E}}_{2}\,\left[n\right]$. This result showed excellent agreement in the time domain between the real and virtualized recordings at both HAD microphones, with MAEs of −82 dB and −84 dB. An MAE of no more than −77 dB was achieved for all loudspeaker positions, and the average MAEs amongst the loudspeaker positions was −81 dB at each microphone. Informal subjective headphone listening tests with five expert listeners found that the reproduced recording was indistinguishable from the target for each loudspeaker position and that the reproduction was free of any time domain artifacts such as echoes or smearing. Binaural audio files of the measured results are available in the Supplementary Material.

#### Clinical Booth

**FIGURE 9:**
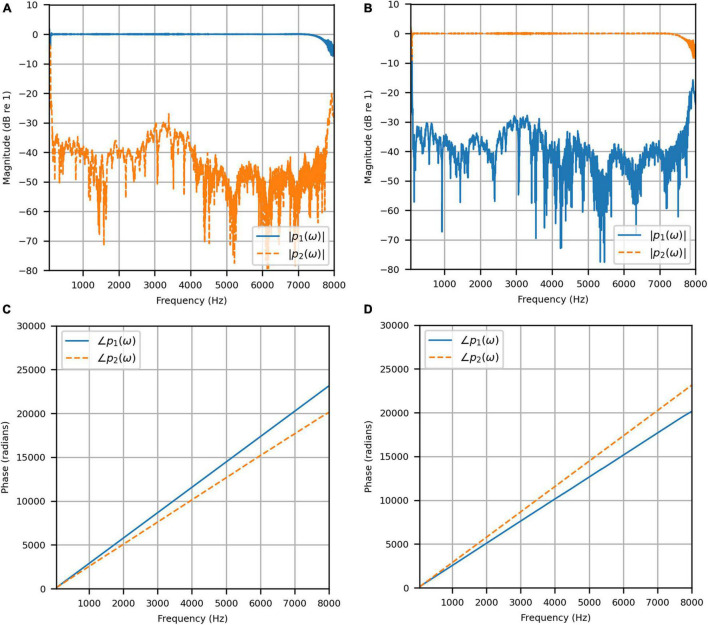
Clinical booth: **(A)** Reproduced magnitude responses for a target impulse to the left microphone (blue solid); **(B)** Reproduced magnitude responses for a target impulse to the right microphone (red dashed); **(C)** Reproduced phase responses for a target impulse to the left microphone (blue solid); **(D)** Reproduced phase responses for a target impulse to the right microphone (red dashed).

**FIGURE 10:**
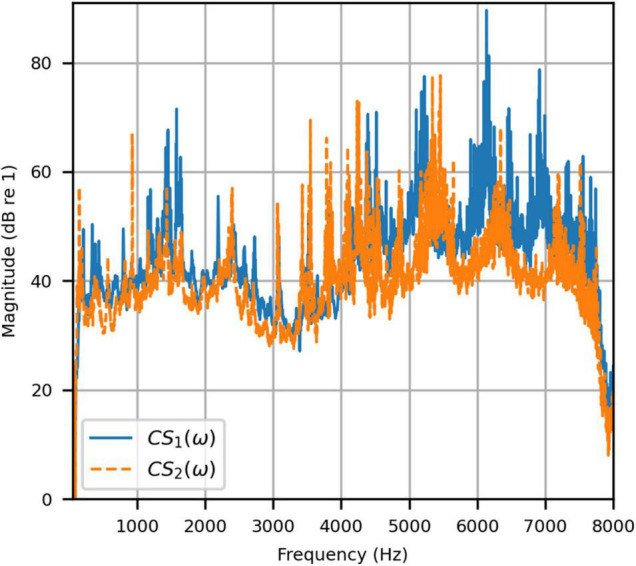
Clinical booth: Channel separations for a target impulse to the left (blue solid) and right (red dashed) microphones.

To attenuate certain adverse room reflections (present only in the clinical booth HADIRs), the temporal window parameters were adjusted for the clinical booth to attenuate later reflections (not present in the anechoic chamber). Figure 9 shows the reproduced magnitude responses |*p*_1_(ω)|,|*p*_2_(ω)|and unwrapped phase responses ∠*p*_1_(ω), ∠*p*_1_(ω)as functions of frequency in Hz for frequencies 50–8000 Hz, again as a result of a target impulse to the left and right input channels. Like the anechoic reproduction, the target impulse signal was reproduced with an almost flat response centered about 0 dB. There were slightly more fluctuations; however, these remained within 1 dB for frequencies above 100 Hz. The opposite channel had been significantly attenuated, although to a lesser extent than seen in the anechoic chamber. Figure 10 shows channel separations as functions of frequency in Hz. Still, there was no less than 25 dB of separation at most frequencies between 100 and 7800 Hz (the effective passband). These results suggested that accurate sound field control at the HAD microphones is possible in the audiological testing booth.

**FIGURE 11:**
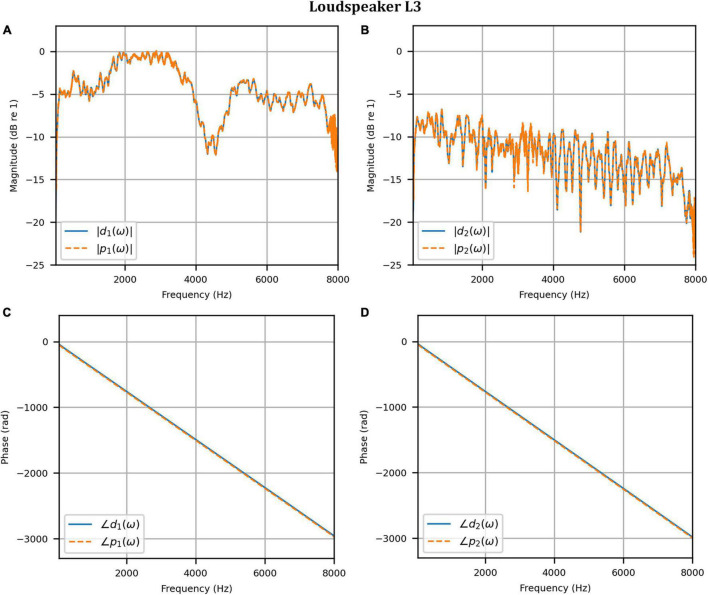
Clinical booth: Target L3 anechoic HADRTF (blue solid) versus reproduced (red dashed) frequency responses: **(A)** Magnitude responses at the left microphone; **(B)** Magnitude responses at the right microphone; **(C)** Phase responses at the left microphone; **(D)** Phase responses at the right microphone.

To reinforce the accuracy of the booth reproduction, and to evaluate the room adaptability potential of the VA system, the target binaural signal was set as the HADRTF measured from the loudspeakers *from within the anechoic chamber*. Thus, the objective was to reproduce a response with essentially no reverberation within a room with reverberation. Figure 11 shows the reproduced magnitude and phase responses at each microphone when the target HADRTF was due to loudspeaker L3. There was excellent agreement in the magnitude responses within the passband, again with only negligible fluctuations below 100 Hz (4 dB at 60 Hz), as predicted by the channel separation analysis. As with the anechoic chamber, the phase responses were reproduced accurately, albeit with a constant shift that retained the inter-microphone phase relationships. Excellent agreement was also observed in the frequency responses for the remaining loudspeaker positions. This result showed that the VA system is capable of room adaption *and* accurate reproduction in a real-world clinical space.

**FIGURE 12:**
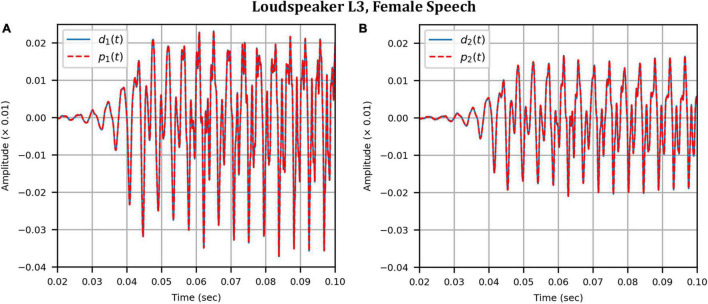
Clinical booth: Target (blue solid) versus reproduced (red dashed) time domain waveforms of a female speech sample played from loudspeaker L3 (originally in the anechoic chamber) reproduced in the audiological booth: **(A)** Left microphone; **(B)** Right microphone.

**FIGURE 13:**
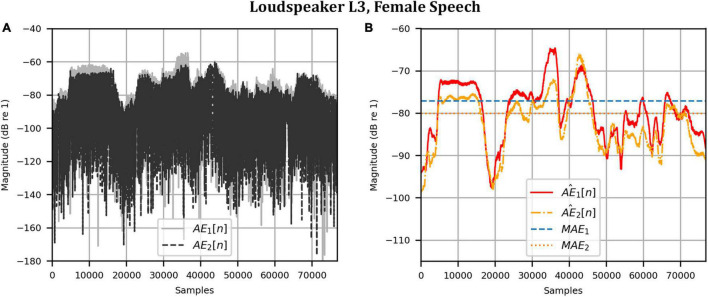
Clinical booth: Error metrics quantifying the difference between the target and reproduced time domain waveforms in Figure 12: **(A)** Unsmoothed absolute errors; **(B)** Smoothed absolute errors and mean absolute errors.

As before, the time domain performance was investigated by comparing the front HAD microphone recordings of the female speech sample played from each loudspeaker, although this time from within the anechoic chamber, to a recording of the VA system’s reproduction of those same target microphone signals using loudspeakers L1 and R1 within the booth. Figure 12 compares a portion of the reproduced versus target (played from loudspeaker L3) time domain waveforms. Again, after removal of the constant delay, excellent agreement between the target and reproduced waveforms was achieved (and was observed for the other loudspeaker positions). Again, as expected from the HADRTF reproduction in the booth, we found that the agreement in the time domain reproduction was excellent for all six loudspeakers. Following the format of Figures 8, 13A shows the unsmoothed AEs and Figure 13B shows the smoothed AEs and MAEs calculated from the time domain signals. This result reinforced the excellent agreement in the time domain between the real and virtualized recordings at both HAD microphones in the clinical booth environment, albeit to a slightly lesser extent than in the anechoic chamber, with MAEs of −77 dB and −80 dB. An MAE of no more than −75 dB was achieved for all loudspeaker positions, and the average MAEs amongst the loudspeaker positions were −77 dB at each microphone (4 dB less than in the anechoic chamber). Again, in informal subjective listening tests with expert listeners, the target and virtualized recordings could not be differentiated for each loudspeaker position (for audio demonstrations, see the Supplementary Material).

### Head Rotation Measurements

For each head rotation angle in both uncompensated and compensated modes of operation, we evaluated the achieved channel separation as a function of frequency, and the AEs and the MAEs (see section “Metrics”). Here, we report only the channel separation and the MAEs for brevity (data for the AEs is provided in the supporting data). For the MAEs (time domain performance), the target audio was the female speech sample (as measured from each loudspeaker in the anechoic chamber). The performance of the system when the target loudspeaker was L3 is highlighted.

#### Anechoic Chamber

**FIGURE 14:**
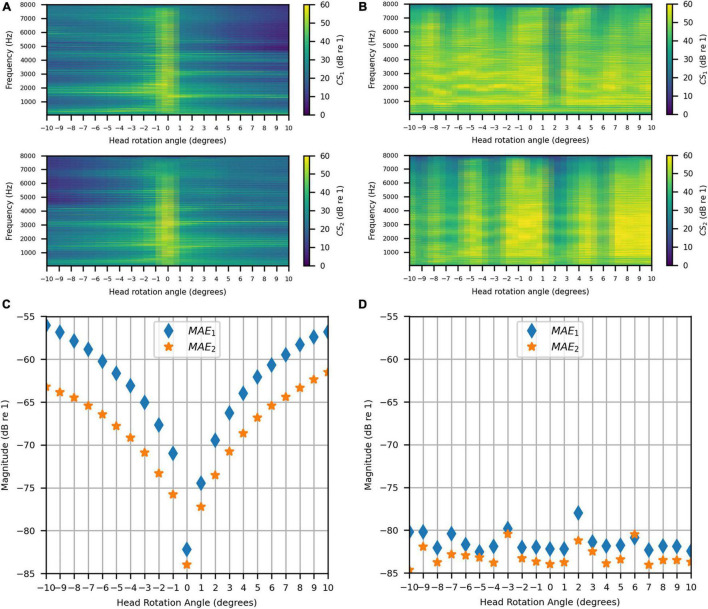
Anechoic chamber: **(A)** Uncompensated channel separation as a function of frequency in Hz and head rotation angle; **(B)** Compensated channel separation as a function of frequency in Hz and head rotation angle; **(C)** Uncompensated mean absolute errors as functions of head rotation angle; **(D)** Compensated mean absolute errors as functions of head rotation angle.

Figures 14A,B show the achieved channel separations measured in the anechoic chamber with and without compensation for head rotation, respectively, as 2D functions of head rotation angle in degrees and frequencies 50–8000 Hz. Figure 14A shows that substantial channel separation was achieved in the passband (greater than 60 dB for some frequencies and at least 20 dB for most frequencies) for head rotation angles of up to 2° to the left or right when the filters were not updated to account for rotation. Beyond 2°, the channel separation lessened as the head rotation angle increased, as expected. However, it still exceeded our target performance of 20 dB or more for many frequencies, despite the lack of filter adaptation. Figure 14B shows that the channel separation was substantially higher (nearly 60 dB or more for many frequencies) and more consistent with increase in head rotation angle when the inverse filters were updated with changes in angle.

Figures 14C,D show the uncompensated and compensated MAEs measured in the anechoic chamber, respectively, as functions of head rotation angle in degrees. Figure 14C shows that the MAEs were lowest (−82 dB and −84 dB) for the centered head position (0°), as expected. The MAEs increased with head rotation angle in either direction, reaching a maximum of −54 dB with the largest head rotation angle (10°). In contrast, Figure 14D indicates that when the filters were updated with rotation, the MAEs stayed consistently below −80 dB for most head rotation angles (except for two outliers at −3° and 2°, which were around −80 dB and −78 dB, respectively, for *MAE*_*1*_). These results show that the system is robust to minor head rotation, but that performance is much better and more consistent (particularly in the time domain) when the filters are updated with head rotation angle.

#### Clinical Booth

**FIGURE 15:**
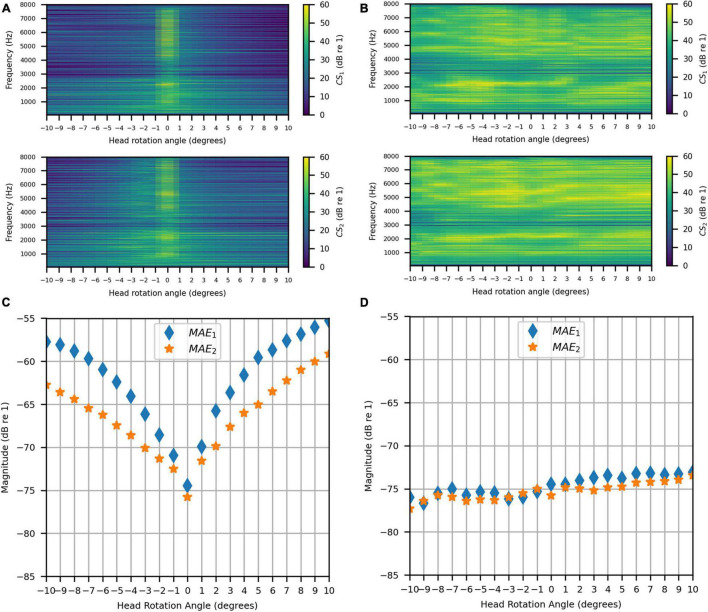
Clinical Booth: **(A)** Uncompensated channel separation as a function of frequency in Hz and head rotation angle; **(B)** Compensated channel separation as a function of frequency in Hz and head rotation angle; **(C)** Uncompensated mean absolute errors as functions of head rotation angle; **(D)** Compensated mean absolute errors as functions of head rotation angle.

The same evaluation was done in the clinical booth as in the anechoic chamber. As for the stationary measurements, the target signal was measured in the anechoic chamber. Figures 15A,B show the uncompensated and compensated channel separations measured in the booth, respectively, as functions of head rotation angle in degrees and for frequencies 50–8000 Hz. Figure 15A shows that, as in the anechoic chamber, significant channel separation was achieved in the passband when the head was not rotated (60 dB or more for some frequencies and as little as 20 dB for most frequencies except for some in the range of 3000–3500 Hz). When the head rotation was 1° to the left, the achieved channel separation was 20 dB or more from around 160 to 2700 Hz. Above 2700 Hz, the right channel separation fluctuated around 20 dB while the left channel separation generally stayed above 20 dB (except for some frequencies in the range of 3000–3600 Hz). For 1° rotation to the right, 20 dB or more of channel separation was generally achieved between 160 and 6000 Hz for both channels. Beyond 1°, in either direction, the channel separation lessened and tended to become worse with increasing angle (being as little as 5 dB at some frequencies between 160 and 2700 Hz at 10° of rotation). For all angles, the channel separation tended to be substantially lower for frequencies above 2700 Hz. However, separation remained substantial (20 dB or more) for some frequency bands. Figure 15B shows that, like for the anechoic chamber, when the inverse filters were updated to compensate for rotation, the channel separation was significantly higher (30 dB or more for many frequencies) and remained more consistent as head rotation angle increased. However, channel separation was reduced in the range of 3000–4000 Hz for all angles.

Figures 15C,D show the uncompensated and compensated MAEs measured in the clinical booth, respectively, as functions of head rotation angle in degrees. Figure 15C shows that the MAEs were lowest (around −75 dB) for 0°, as expected. However, MAEs increased with head rotation angle in either direction, reaching a maximum on each side with the largest head rotation angle. Note that the MAE for 0° was slightly less than reported in the previous section on stationary measurements (see sections “Stationary Measurements” and “Clinical Booth”). This is likely due to slight alterations in the room acoustics caused by differences in placement of the clinical equipment used in the test booth (which is part of an active clinic). Figure 15D indicates that, like for the anechoic chamber, when the filters were updated with rotation, the MAE stayed consistently below −70 dB for most head rotation angles. A slight asymmetry was observed where the error was higher for positive angles, which was likely due to asymmetric room reflections.

## Discussion

We investigated the ability of a two-loudspeaker VA system to control HAD microphone pressure signals using CTC both in an anechoic chamber and a representative audiological testing booth. Our system has a small physical footprint and low cost compared to previous approaches, which required large loudspeaker arrays (e.g., Kitterick et al., 2011; Grimm et al., 2016). We evaluated the proposed VA system by attempting to reproduce target signals at the front microphones of behind-the-ear HADs worn by a KEMAR head and torso simulator. Using our proposed inverse filter design process, we showed a high reproduction accuracy under anechoic conditions, both in the time and frequency domain, when the head and torso simulator was kept still. To demonstrate the performance in a real-world environment, we repeated this stationary evaluation process in an audiological testing booth with only moderate acoustic treatment. We showed that the VA system performance within the booth was comparably accurate to the anechoic-based reproduction for the same anechoic target signals. In both spaces, we showed that the achievable channel separation in the passband matched, and for some frequencies exceeded, our target performance of 20 dB, which is the reported minimum needed to accurately reproduce the perception of the intended binaural signals for normal-hearing listeners. These findings demonstrate that the VA system can overcome the room acoustics within the testing booth. These results establish a baseline physical performance standard against which alternative systems and approaches can be assessed.

Head-related transfer functions can change markedly even with quite small head movements (Yu et al., 2018). Because of this, in clinical settings, participants are typically instructed to not move their heads. Nonetheless minor head movement (postural sway) is expected (e.g., Hirahara et al., 2010; Denk et al., 2017). Therefore, we investigated the effect of head rotation on the system’s performance when using inverse filters designed assuming a stationary forward-facing listener (i.e., uncompensated performance). We compared the uncompensated performance to the performance when inverse filters were updated to account for head rotation (i.e., compensated performance). We found that the system was robust to minor head rotations of around 1° or 2° without compensation. However, for larger head rotations (3° or more) notable deviations from the target performance were observed. However, a psychophysical evaluation has not yet been conducted so it is unclear how much these deviations affect the perceived binaural signals. Hirahara et al. (2010) showed that participants who were instructed to stay still during HRTF measurements exhibited postural sway of no more than 2° for 5 minutes of continuous testing, which increased to between 3° and 5° (depending on the subject) after 20 minutes of continuous testing. Denk et al. (2017) showed that postural sway could be reduced to about 0.5° when the listener was given visual feedback on their current head position and allowed to realign before each measurement. A similar visual feedback system could be implemented during VA system measurements to reduce head movement during testing.

We showed that, when head rotation was compensated for, the performance was comparable to the baseline forward-facing and stationary performance across the full range of angles tested. This suggests that a dynamic system that adapts to listener position could markedly improve performance. In our measurements, the inverse filter design parameters, such as regularization and windowing applied to the HADIRs, were not varied with head rotation angle, and thus there is scope to further optimize these parameters. This may particularly benefit performance in the clinical booth, where room reflections influenced physical outcomes. It should be noted that the approach for compensating head movements that we presented requires either prior knowledge of the HADIRs for a given head rotation (e.g., Gálvez et al., 2019) or real-time measurement of HADIRs and updating of inverse filters (e.g., Kabzinski and Jax, 2019). An adaptive approach could be used with direct access to the HAD microphone signals and could allow for more listener movement during measurement, assuming the rate of head movement is not rapid (Kabzinski and Jax, 2019). Alternatively, a head tracking system could be used to reject the small number of trials where excessive head rotation occurs (e.g., Denk et al., 2017). In addition to compensating for head movement, future work should establish how effective these adaptive techniques are for different HADs, as well as for different head and pinna shapes and sizes.

The measured performance of the VA system suggests it has strong potential for clinical use. The high reproduction accuracy shows that the system can reproduce complex spatial auditory scenes. It could therefore improve diagnostic testing and assessment of HAD signal-processing performance by greatly increasing the potential for ecologically valid tests. Furthermore, its small footprint and low cost mean that it could find widespread use, including across low- and middle-income countries. While we used custom HADs with direct microphone access, which aren’t currently commercially available, this access to the microphone signals could be gained using Bluetooth Low Energy. Bluetooth Low Energy is already used in most of the latest hearing-assistive devices and is capable of simultaneous multichannel output streaming. This existing technology could readily be adapted to allow audio streaming from device microphones to a VA system. However, the latency of the Bluetooth Low Energy transmission is a potential limitation that should be explored for its use in the system, especially in an adaptive mode of operation. If access to the microphone cannot be gained then additional measurements and equipment would be required (as in Chan et al., 2008; see section “Introduction”), which could be both time consuming and make the system more expensive.

Future work is required to fully establish the efficacy of our proposed approach for use in clinical audiology. While we have demonstrated accurate sound field control at two microphones, it remains to be shown how well the system can control the pressure signals for multiple, closely spaced microphones on a single HAD. Evaluating control at multiple device microphones is important as many modern HADs utilize onboard beamforming algorithms that rely on microphone arrays (e.g., Simon et al., 2020). Future work should evaluate simultaneous sound field control when there are two microphones per device (matching the configuration of many current hearing aids and cochlear implants). Simultaneous control at the ear canals is also desirable, as many HAD users have some degree of residual hearing (Pausch et al., 2018). Proper control at more than two microphones would require the number of loudspeakers to at least match the number of microphones. Thus, at least four loudspeakers would be required to control the sound field at two microphones per device, with two more loudspeakers (six in total) if residual hearing is to also be controlled. A greater number of loudspeakers could have the additional benefit of allowing sound field control at each site to be improved due to the increased focusing capabilities (Hamdan and Fazi, 2021a,b). While this increases the cost of the system, the VA loudspeakers could be housed in a compact enclosure (e.g., a sound bar) and therefore the system’s small footprint could be retained.

A further challenge for the VA system is the influence of visual cues in testing. Since the VA system recreates virtual sound images in directions where there is no loudspeaker or other visual marker, it may be difficult for a listener to properly indicate where different sounds are originating from and performance may be biased toward the VA system loudspeaker array (e.g., Witten and Knudsen, 2005; Mendonça, 2020). There are several potential ways to collect participant responses with the VA system. One would be to use simple markers placed at different locations in the testing room and another would be to deploy head or hand tracking and instruct the participant to direct their head or hand toward the sound source after each trial. To reduce visual biasing effects, VA system loudspeakers could be placed outside of the field of view (e.g., placed laterally) or be disguised (e.g., built into the wall of the testing booth). A more sophisticated approach would be to deploy a virtual or augmented reality headset. This could both give participants a range of visual targets through creation of a custom visual field and allow control of visual biasing effects. These headsets are relatively non-intrusive, low-cost, and would allow the system to maintain a small footprint.

Future work should also establish the link between physical performance and perception of virtual sounds in the clinical environment with HAD users. So far, we have presented a baseline physical performance that was only informally verified perceptually by normal-hearing listeners when listening to the reproduced signals over headphones. Future work should objectively evaluate the perceptual quality of the VA system with HAD users to establish explicit links between the physical and psychoacoustic domains within the intended user groups. Previous work evaluated the amount of channel separation needed for normal-hearing listeners to properly perceive the intended binaural signal using the proposed signal processing approach (e.g., Parodi and Rubak, 2011), however, more information is needed to determine the physical quantities needed for the intended perception in HAD user populations. Future studies should establish, for example, whether virtual sources produce an accurate perception of the intended source location (for both stationary and moving sources), source width, and that unintended spectral coloring doesn’t occur for different source locations or different combinations of virtual sound sources. Study of the effectiveness of sound field reproduction for sources behind the listener might be a particular focus, as CTC systems have typically struggled to effectively reproduce such sources (although this reproduction issue may be reduced as the HAD receiver is behind the ear and therefore subject to less extreme front-back spectral differences). Finally, study of the variability of these precepts between individuals will also be critical. In addition to validating the system, study of the link between the physical signal reproduction and the perceived sound source could lead to a more efficient and simple reproduction system if less physical accuracy were required than previously thought.

Finally, future work should assess the performance of the VA system across a wider range of clinical testing facilities, including those without acoustic treatment. The proposed method has the potential to adapt to non-ideal testing environments that have poor acoustic treatment. Advanced machine learning techniques for acoustic scene classification, such as convolutional neural networks, could be explored to aid effective room adaptation (e.g., Valenti et al., 2017; Zhang et al., 2020). The ability to effectively adapt to a wide range of non-ideal settings is likely to be especially important for supporting clinical audiology in low- and middle-income countries, where acoustically treated facilities are often not available. Effective room adaptation might also open the possibility of using the system in people’s homes. This could allow more advanced remote audiological testing, training, and rehabilitation, which would be highly timely given the recent surge in interest in telemedicine. Furthermore, if shown to be effective at removing the impact of room acoustics (which can reduce intelligibility) in a home environment, this technology could widen access to media and entertainment for hearing-impaired individuals.

## Data Availability Statement

The datasets presented in this study can be found in online repositories. The names of the repository/repositories and accession number(s) can be found below: doi: 10.5258/SOTON/D2081.

## Author Contributions

EH: conceptualization, formal analysis, methodology, investigation, manuscript writing, and funding acquisition. MF: conceptualization, methodology, manuscript writing, project administration, supervision, and funding acquisition. Both authors contributed to the article and approved the submitted version.

## Conflict of Interest

The authors declare that the research was conducted in the absence of any commercial or financial relationships that could be construed as a potential conflict of interest.

## Publisher’s Note

All claims expressed in this article are solely those of the authors and do not necessarily represent those of their affiliated organizations, or those of the publisher, the editors and the reviewers. Any product that may be evaluated in this article, or claim that may be made by its manufacturer, is not guaranteed or endorsed by the publisher.
