# Supplementary material for: A Compact Two-Loudspeaker Virtual Sound Reproduction System for Clinical Testing of Spatial Hearing With Hearing-Assistive Devices
Source: Front Neurosci. 2022 Jan 28;15:725127. doi: 10.3389/fnins.2021.725127 (PMC8835348; doi:10.3389/fnins.2021.725127)
Supplement: Supplementary file 1 [file Data_Sheet_1.PDF]

Supplementary Material

Inverse Filter Design Pseudocode (assuming calculation in Python or Matlab)

% Acquire HADIRs from your chosen method of transfer function acquisition. We used a modified version of the exponential sine sweep proposed by Farina (2000).

% $\boldsymbol{G}_t$ is the $N \times M \times L$ matrix of measured HADIRs

% N is number of time samples of the HADIRs, M is the number of microphones, L is the number of loudspeakers. Here $M = L = 2$.

1. % Normalize the HADIRs:

   $$\boldsymbol{G}_t = \boldsymbol{G}_t / \max\left(\mathrm{abs}(\boldsymbol{G}_t)\right)$$

   % where $\mathbf{max}()$ is the maximum of all matrix elements, $\mathbf{abs}()$ is the absolute value function

2. % Window normalized HADIRs with window vector $\boldsymbol{w}$ (with dimension $N \times 1$):

   For each HADIR in $\boldsymbol{G}_t$:

   $$\boldsymbol{G}_t[:, i, j] = \boldsymbol{G}_t[:, i, j] * \boldsymbol{w}$$

   % where $i = 0, 1, \ldots, M$ and $j = 0, 1, \ldots, L$

   % * denotes elementwise multiplication between vectors

3. % Calculate regularized pseudoinverse:

   % First, convert HADIRs to frequency domain transfer functions $\boldsymbol{G}$ using Discrete Fourier Transform (DFT):

   $$\boldsymbol{G} = \mathrm{fft}(\boldsymbol{G}_t)$$

   % keeping only the first (N/2)+1 (if N is even) or (N+1)/2 (if N is odd) frequencies of the full DFT

   % $\boldsymbol{G}$ has dimensions $K \times M \times L$, where K is the number of discrete frequencies

   % Next, calculate Tikhonov regularized pseudoinverse matrix $\boldsymbol{H}$ (with dimension $K \times L \times M$):

   For each frequency k:

   Let $\boldsymbol{G}^H[k, :, :] = \mathrm{transpose}\left(\mathrm{conj}(\boldsymbol{G}[k, :, :])\right)$

$$\boldsymbol{H}[k,:,:] = \boldsymbol{G}^H[k,:,:] \text{ @ } \mathrm{inv}(\boldsymbol{G}[k,:,:]\boldsymbol{G}^H[k,:,:] + \beta * \boldsymbol{I})$$

% where **tranpose**() denotes the matrix transpose, **conj**() denotes complex conjugation, **inv**() denotes the matrix inverse, and **@** denotes matrix multiplication

% β is the scalar Tikhonov regularization parameter, $\boldsymbol{I}$ is a $M \times M$ identity matrix

4. % Lowpass inverse filters:

% First, convert frequency domain inverse filters to time domain filters $\boldsymbol{H}_t$ (with dimension $N \times L \times M$) using inverse DFT:

$$\boldsymbol{H}_t = \mathrm{ifft}(\boldsymbol{H})$$

% Next, create lowpass filter coefficients $\boldsymbol{lowpass}$. We used **firwin()** and **lfilter()** from the Python module scipy.signal (Version 1.6.2) to generate the lowpass and apply it, respectively.

$$\boldsymbol{lowpass} = \mathrm{firwin}(99, 8000,\ 'lowpass', 48000)$$

% The lowpass filter was a linear phase FIR with 99 taps and cutoff frequency set at 8000 Hz, sampling rate was 48000 Hz

$$\boldsymbol{H}_t = \mathrm{lfilter}(\boldsymbol{lowpass}, 1.0, \boldsymbol{H})$$

5. % Shift and normalize inverse filters

% We used the Python library NumPy (Version 1.20.2) to apply its **roll()** function to shift the time domain inverse filters after applying the lowpass

$$\boldsymbol{H}_t = \mathrm{roll}(\boldsymbol{H}_t, T)$$

% where T is the shift amount in samples

% Finally, we normalized the inverse filters:

$$\boldsymbol{H}_t = \boldsymbol{H}_t / \max\left(\mathrm{abs}(\boldsymbol{H}_t)\right)$$

References

Farina, A. (2000). Simultaneous Measurement of Impulse Response and Distortion with a Swept-Sine Technique. Audio Eng Soc Conv 108, 5093. http://www.aes.org/e-lib/browse.cfm?elib=10211
